# T-cell immunoglobulin and mucin-domain containing-3 orchestrates myeloid dysfunction across brain aging and disease

**DOI:** 10.4103/NRR.NRR-D-25-01348

**Published:** 2026-01-02

**Authors:** Berta Segura-Collar, Lucia Mondejar-Ruescas, Carmen Jareño-Bonilla, Ricardo Gargini

**Affiliations:** Instituto de Investigaciones Biomédicas I+12, (Imas12) Hospital 12 de Octubre, Madrid, Spain; Dpto Anatomía Patológica, Hospital 12 de Octubre, Madrid, Spain; Dpto Oncología Médica, Hospital 12 de Octubre, Madrid, Spain

The old definition of the central nervous system as an “immune privilege” site is now considered obsolete, as it has been demonstrated that there is a complex interaction between immune cells and other brain cells, which creates an environment-controlled surveillance with different immune niches that are now beginning to be understood. Thus, brain aging is characterized by a series of immunological, vascular, and metabolic changes that erode defense mechanisms and neuronal homeostasis of the neural circuits. Among the most notable is inflammaging, defined as a basal state of low-grade chronic inflammation that progressively sets in and affects synaptic function, neuronal plasticity, and cell viability. This proinflammatory environment is accompanied by dysregulation of resident immune cells, especially in microglia and brain macrophages, which progressively transition from a homeostatic phenotype to a regulatory-immunosuppressive state (Segura-Collar et al., 2025). The progressive loss of integrity of the blood-brain barrier is a key event in this process, as it allows the entry and persistence of peripheral immune cells with the ability to adapt to the brain microenvironment, thereby accelerating the onset and chronicity of neurodegenerative and neoplastic diseases (Segura-Collar et al., 2022).

One the most relevant changes identified in the last decade is the progressive imbalance between inflammatory and regulatory circuits in the central nervous system (CNS), caused by the colonization of peripheral myeloid cells which, upon accessing aged or injured brain tissue, acquire an immunosuppressive profile characterized, among other markers, by the expression of T-cell immunoglobulin and mucin-domain containing-3 (TIM-3) (encoded by HAVCR2 in humans), co-inhibitory receptor expressed on multiple immune cell types in both the periphery and the CNS. In addition to being expressed on effector T cells (interferon-γ producers), regulatory T cells (FoxP3^+^), exhausted T cells, and tumor-infiltrating lymphocytes, TIM-3 has also been found on innate myeloid cells, including CNS-resident microglia, dendritic cells, and other infiltrating myeloid populations, such as monocyte-derived macrophages, generating an immunosuppressive microenvironment through binding to its ligands. For instance, the binding of TIM3 to galectin-9 induce apoptosis on T cells and reducing their proliferation and cytokine production. Interaction with CEACAM1, co-expressed on T cells and myeloid cells (DCs, macrophages), promotes immune tolerance and T cell exhaustion. HMGB1 binds to TIM-3 on dendritic cells limiting the immune response. Finally, PtdSer, exposed on apoptotic cells, facilitates antigen cross-presentation by TIM-3, contributing to immune regulation and tolerogenic processes that suppress and modulate immune responses in both peripheral tissues and the CNS (Zhang et al., 2024).

**Figure 1:**
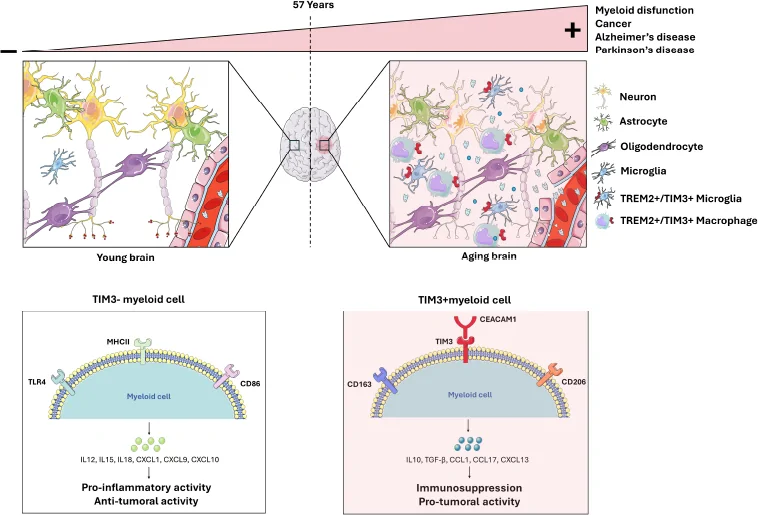
Representative schematic diagram summarizing the principal findings of this paper. Brain aging is accompanied by a progressive loss of neuronal synaptic connections and sustained neuroinflammatory responses. This chronic inflammatory milieu promotes the expansion and activation of pathological myeloid cell subsets characterized by TREM2 and TIM3 expression, which accumulate within the central nervous system and contribute to neurodegenerative and tumoral processes. Image provided by Servier Medical Art (https://smart.servier.com), licensed under CC BY 4.0 (https://creativecommons.org/licenses/by/4.0/). CCL: CC chemokine ligand; CXCL: CXC chemokine ligand; IL: interleukin; TGF: transforming growth factor; TIM3: T-cell immunoglobulin and mucin-domain containing-3; TLR4: Toll-like receptor 4.

Notably, upregulation of TIM-3 in myeloid populations becomes more pronounced with aging and in pathological states, positioning TIM-3 expression as a central hallmark of brain tolerogenesis and a major mechanism of resistance to both conventional and emerging treatments (Zhang et al., 2024). This mechanism is observed in a variety of pathological contexts, such as Alzheimer’s disease, Parkinson’s disease, and glioblastoma, representing a shared vulnerability of the brain to degeneration and cancer (**Figure 1**; Kimura et al., 2025; Segura-Collar et al., 2025; Yin et al., 2025). Recent studies have convincingly demonstrated that these TIM-3^+^ myeloid cells are not only biomarkers of immune dysfunction, but also actively remodel the brain microenvironment, promoting immune tolerance, loss of synaptic surveillance, and enhanced tumor immune evasion (Segura-Collar et al., 2025), process by which tumor cells avoid detection and destruction by the immune system through intrinsic and extrinsic mechanisms, such as the expression of inhibitory molecules or suppression of surrounding immune cells, respectively. This commentary expands and contextualizes these observations by incorporating recent contributions on the regulation of microglia in the tumor environment and the consequences of this interaction on the progression of neurological pathologies, while also integrating the latest advances reported in these articles. It also seeks to synthesize the cellular and molecular mechanisms involved, highlight the pathological importance of these processes, and explore emerging therapeutic proposals.

**Microglia and myeloid cells — TIM-3**^**+**^ **as orchestrators of brain immune remodeling: *Microglia in aging — Functional plasticity and immune reprogramming***

Microglia constitute the core of resident immune system of the brain and display remarkable functional plasticity shaped by microenvironmental signals, including neuronal activity, the presence of tissue damage, and exposure to circulating factors. Under physiological conditions, they contribute to synaptic surveillance, debris removal, neural circuit remodeling, and preservation of homeostatic maintenance (Paolicelli et al., 2022). However, with aging or in the contexts of persistent tissue damage, microglia undergo reparative functions and acquire chronic reactivity properties and phenotypes with immunosuppressive predominance (Paolicelli et al., 2022). Among the molecular mechanisms that drive the shift, TIM-3 signaling stands out, which reinforces transforming growth factor β/SMAD2 [mothers against decapentaplegic homolog 2] cascades, inducing a state of quiescence and suppression of innate adaptive immune responses (Kimura et al., 2025).

Evidence from genetically engineered models supports this concept: selective deletion of TIM-3 in microglia generates a paradoxical phenotype that is less tolerogenic, more resistant to disease-associated states, and more efficient at clearing amyloid deposits in Alzheimer’s disease models. This phenotype preserves neurocognitive performance and sustains synaptic plasticity (Kimura et al., 2025). Importantly, microglial suppression also influences neuron–tumor interactions, facilitating glioma progression by two complementary mechanisms: (i) neuronal secretion of pro-tumor factors, and (ii) the generation of signals from neuron–tumor crosstalk that reinforce myeloid dysfunction.

Collectively, these findings highlight TIM-3 as a pivotal checkpoint that locks microglia into an immunosuppressive state. While this may be beneficial in acute settings, its chronic activation compromises the ability of the brain to eliminate pathogens, misfolded proteins, and malignant cells, ultimately fostering neurodegeneration and tumor development (**Figure 1**).

***Peripheral myeloid cell infiltration and disruption of brain homeostasis:*** The alteration of the blood–brain barrier during aging allows the infiltration of circulating monocytes, which subsequently differentiate in situ into TREM2^+^/TIM-3^+^ brain macrophages with a regulatory phenotype (Segura-Collar et al., 2025). Our recently published results show that, from the age of 57 onwards, the accumulation of myeloid cells derived from monocytes increases, coinciding with vascular deterioration and the appearance of microenvironments of chronic inflammation rich in transforming growth factor β (Koh et al., 2015; Segura-Collar et al., 2025). Once established, these infiltrating cells expand locally and contribute to myeloid dysfunction in the context of neurodegeneration, high- and low-grade brain tumors, and pediatric tumors with key epigenetic alterations (such as histone H3 mutation) (Ausejo-Mauleon et al., 2023; Segura-Collar et al., 2025).

One of the main consequences of TIM-3^+^ myeloid cells colonization is the establishment of a local immunosuppressive niche, which facilitates tumor immune escape, synaptic atrophy, and decreased response to infections, signs of damage, or malignant transformation. In glioblastoma, for example, a higher density of functional connections between the tumor and healthy neural networks predicts a poorer prognosis and accelerated cognitive decline. This effect is partly explained by the ability of these cells to modulate neuronal activity, lymphocyte infiltration, and the composition of the tumor microenvironment (Krishna et al., 2023; Nejo et al., 2025).

***Glioblastoma-induced synaptic and functional remodeling:*** Recent advances in intracranial neurophysiology and single-cell transcriptomics have revealed that gliomas, particularly glioblastoma, are capable of functionally integrating into and remodeling human neural networks. This process alters not only local excitability but also the synaptic architecture and immune landscape of infiltrating regions, reshaping between neuronal activity and immune surveillance.

Glioblastoma establishes direct synaptic contacts with neurons, responding to neuronal activity and promoting its own growth through paracrine signaling and functional synapses. In highly functionally connected intratumoral regions, tumor cells express elevated levels of synaptogenic gene expression—notably thrombospondin-1 (TSP-1). This factor, which is secreted in a healthy brain by astrocytes to promote formation of new synapses, is expressed by tumor cells and myeloid cells in the highly functionally connected regions of high-grade gliomas, facilitating the tumor integration into neural circuits essential for cognition. Consequently, neural circuits are remodeled, accelerating tumor growth and impairing the patient’s cognitive performance, which significantly shortens their survival (Nejo et al., 2025)

Concomitantly, resident microglial and infiltrating macrophages respond to TSP-1-mediated synaptic remodeling by promoting the consolidation of a tolerogenic and anti-inflammatory environment, which restricts antigen-presenting cell activity and reduces effector T lymphocytes infiltration. The TSP-1 → synaptic remodeling → regional immunosuppression axis has been established as the central mechanism of tumor escape and aggressive progression, particularly in tumors with a high degree of integration into neural circuits (Krishna et al., 2023; Nejo et al., 2025).

***From partners to accomplices — microglia and macrophages in tumor progression:*** Microglia and perivascular macrophages normally cooperate to maintain neuronal homeostasis by regulating synaptic activity and supporting the neurovascular unit (Segura-Collar et al., 2022). However, spatial and transcriptomic analyses have revealed that in highly connected tumor regions, the proportion of myeloid cells, particularly monocyte-derived tumor-associated macrophages, with anti-inflammatory and immunosuppressive phenotypes is markedly increased. These cells express genes such as *CD163*, *LGALS1/3*, *RNASE1*, *TSP-1*, and, critically, *TIM-3*, which enhances their capacity to inhibit proinflammatory signaling and antigen-presenting functions (Nejo et al., 2025; Segura-Collar et al., 2025). By contrast, in regions of low connectivity, proinflammatory profiles predominate, with higher expression of genes linked to antigen presentation and immune activation.

The functional plasticity of microglia and macrophages is, therefore, not merely a response to tissue damage or inflammation, but is actively orchestrated by the tumor and modulated by neuronal activity. This dynamic creates a vicious cycle that fuels glioblastoma progression while progressively undermining CNS integrity and function.

**Neuroimmune crosstalk —TIM-3**^**+**^ **myeloid cells, synaptic remodeling, and disease progression:** The chronic establishment of an immunosuppressive microenvironment in the aging brain promotes the accelerated progression of both neurodegenerative disorders and brain tumors once homeostatic balance is disrupted. TIM-3^+^ myeloid cells and tolerogenic microglia play a central role in orchestrating this milieu by suppressing antigen presentation, limiting cytotoxic T cell infiltration, and promoting programmed death (PD)-1/PD-L1–mediated T cell exhaustion, thereby impairing both innate and adaptive immune responses (Segura-Collar et al., 2025).

In Alzheimer’s and Parkinson’s disease models, this phenotype reduces the clearance of aggregated proteins such as amyloid-β and alpha-synuclein, facilitating the spread of neurodegeneration and the loss of connectivity between key brain regions. Tolerogenic microglia further decrease physiological synaptic pruning, interfering with synaptic plasticity and learning, thereby compounding cognitive and functional decline (Paolicelli et al., 2022).

Similarly, in high-grade gliomas with extensive tumor–neural connectivity, this immunosuppressive environment accelerates tumor progression, reduces adaptive immune responses, and shortens patient survival, illustrating a shared pathogenic mechanism between neurodegeneration and brain tumors (Nejo et al., 2025). Tumors with higher neuronal integration exhibit more pronounced immune evasion and worse prognosis compared with less integrated tumors. The failure to mount targeted inflammatory responses against pathogens, misfolded proteins, or transformed cells underlies both functional decline and poor therapeutic outcomes (Segura-Collar et al., 2025).

The neuroimmune axis further contributes to tumor progression in both central and peripheral cancers, including prostate, head and neck, and gastric malignancies. Neuronal death triggers interleukin-6 and tumor necrosis factor α release, which in turn induce local immunosuppression and myeloid dysfunction, compromising antitumor immunity (Restaino et al., 2025). Spatial transcriptomic analyses in humans and murine models reveal an inverse correlation between synaptic gene expression and immune response pathways, including tumor necrosis factor α/nuclear factor κB signaling, interferon response, and other proinflammatory networks. In essence, the greater the neuronal integration of tumors, the lower the local inflammation and immune surveillance capacity, highlighting a key mechanism by which tumor synaptogenesis facilitates immune evasion and aggressive tumor behavior (Nejo et al., 2025).

Collectively, these findings define a unifying model in which TIM-3^+^ myeloid cells and tolerogenic microglia establish a shared axis of immune suppression and synaptic remodeling, driving the progression of both neurodegenerative diseases and gliomas. This model highlights the interconnection between immune dysfunction and neural circuit disruption and identifies TIM-3^+^ myeloid populations as promising targets for therapeutic intervention.

**Emerging therapeutic strategies — restoring immune surveillance and brain function:** The convergence of aging-related alterations, vascular dysfunction, chronic inflammation, and expansion of TIM-3^+^ myeloid populations help explain the pronounced resistance of brain tumors, particularly high-grade gliomas, to spontaneous immune responses and conventional immunotherapies. The predominance of a microenvironment characterized by low antigen presentation, high TIM-3 myeloid cell activity, elevated TSP-1 expression, and reduced cytotoxic T lymphocyte infiltration effectively suppresses tumor recognition and destruction, creating a formidable barrier to therapy.

***TIM-3-blockade and microglia reprograming:*** Preclinical studies have explored multiple approaches to counteract TIM-3-mediated immunosuppression. In pediatric brain tumors (such as diffuse intrinsic pontine glioma), the administration of TIM-3 blocking antibodies reprogram myeloid cells toward a proinflammatory phenotype, increasing T-cell infiltration and restoring antigen presentation, and generating durable tumor responses with evidence of immunological memory (Ausejo-Mauleon et al., 2023). Combined TIM-3 and PD-1 blockade further reactivates myeloid-mediated antigen presentation in the aging brain, preventing tumor development (Segura-Collar et al., 2025).

In adult glioblastoma models, inhibition of TSP-1 — genetically (knockout) or pharmacologically through agents such as gabapentin or perampanel that modulate glutamatergic pathways — reduces tumor integration into the neuronal circuit, reprograms the function of microglia/macrophages to less immunosuppressive states, increases CD8⁺ T-cell infiltration, and prolongs survival (Nejo et al., 2025). Normalization of the blood-brain barrier additionally limits the recruitment of dysfunctional peripheral myeloid cells, restoring protective immune surveillance circuits (Segura-Collar et al., 2025).

***Enhancing efficacy through adaptive immunotherapy:*** While TIM-3 or TSP-1 inhibition alone demonstrates promising effects, combination strategies — including CAR-T cells and checkpoint inhibitors, a class of immunotherapy drugs that block checkpoint proteins expressed by tumor or immune cells to prevent their destruction or inhibition by the immune system (e.g., anti-PD-1) — further enhance immune reactivation and improve tumor control in animal models (Segura-Collar et al., 2025). While these findings still need to be consolidated in clinical trials, modulation of the microglia/myeloid-TIM-3 axis is a critical step in overcoming the inherent immunoresistance observed in the CNS, which has historically limited the efficacy of immunotherapy in the brain.

***Aging, immune escape, and therapeutic opportunities:*** Accumulating evidence supports a new paradigm in neurology and neuro-oncology: brain aging is inseparable from profound immune remodeling, where microglia, macrophages, and other central and peripheral myeloid cells, modulated by TIM-3 and TSP-1, constitute both signatures of vulnerability and an active regulator of homeostasis, synaptic plasticity, and tumor surveillance.

Understanding and manipulating these immunoregulatory circuits will not only enable the design of more precise therapies against tumors but also improve the response to treatments for dementia and other neurodegenerative diseases, opening the door to strategies for prevention and cerebral immune rejuvenation.

**Conclusion:** The aging–vascular dysfunction–microglial remodeling axis emerges as the central determinant of brain vulnerability to both neurodegeneration and cancer. With the advent of spatial transcriptomics, high-resolution electrophysiology, and integrative modeling, it is now possible to delineate how TIM-3^+^/TSP-1^+^ myeloid cells orchestrate the immunosuppressive landscape that fuels tumor progression and accelerates functional decline.

Mapping the “personalized synaptic–immune fingerprint” of each brain, and tailoring interventions based on microglial phenotype, tumor–neural connectivity, and vascular health, could inaugurate a new era in prediction, prevention, and precision therapy for CNS diseases.

Recent advances highlight the urgency of translational strategies capable of reprogramming these circuits, with TIM-3 blockade, TSP-1 inhibition, and vascular restoration as pivotal elements. Only by targeting this axis can we aspire to change the natural course of glioblastoma and other age-related pathologies that have long been considered intractable.

*We thank all members of the Neuro-oncology Group and UNMO, the Pathological Anatomy Service and IMAS12 Core Facilities for their excellent assistance. The Neuro-oncology Group and UNMO provided multidisciplinary support for the clinical understanding of brain pathologies, as well as assisting in patient recruitment, clinical data management, and sample collection when necessary. Furthermore, the Pathology Department contributed its diagnostic expertise, including histopathological evaluation and sample processing. Their collaborative work was essential to ensuring a comprehensive perspective.*

*We also thank the Fundacion Blanca Morell for the financial support they provide to the laboratory to carry out our research.*

*This work was funded by ISCIII and FEDER funds: (CP21/00116 and PI22/01171) (to RG), and (CP24/00062) (to BSC).*
